# Crystal structures of 4-(pyrimidin-2-yl)piperazin-1-ium chloride and 4-(pyrimidin-2-yl)piperazin-1-ium nitrate

**DOI:** 10.1107/S1600536814020169

**Published:** 2014-09-13

**Authors:** Thammarse S. Yamuna, Jerry P. Jasinski, Manpreet Kaur, Brian J. Anderson, H. S. Yathirajan

**Affiliations:** a Department of Studies in Chemistry, University of Mysore, Manasagangotri, Mysore 570 006, India; b Department of Chemistry, Keene State College, 229 Main Street, Keene, NH 03435-2001, USA

**Keywords:** crystal structure, pyrimidine–piperazine heterocyclic salts, chloride salt, nitrate salt, bifurcated hydrogen bonds

## Abstract

The title salts, C_8_H_13_N_4_^+^·Cl^−^, (I), and C_8_H_13_N_4_^+^·NO_3_^−^, (II), contain linked pyridinium–piperazine heterocycles. In both salts, the piperazine ring adopts a chair conformation with protonation at the N atom not linked to the other ring. In the crystal of (I), weak N—H⋯Cl inter­actions are observed, leading to zigzag chains along [100]. In the crystal of (II), both H atoms on the NH_2_^+^ group form bifurcated N—H⋯(O,O) hydrogen bonds. Weak C—H⋯O inter­actions are also observed. These bonds collectively link the components into infinite chains along [100].

## Abstract 2

The title salts, C_8_H_13_N_4_^+^·Cl^−^, (I), and C_8_H_13_N_4_^+^·NO_3_^−^, (II), contain linked pyridinium–piperazine heterocycles. In the crystal of (I), weak N—H⋯Cl inter­actions lead to zigzag chains along [100] while in the crystal of (II), bifurcated N—H⋯(O,O) hydrogen bonds and weak C—H⋯O inter­actions collectively link the components into infinite chains along [100].

## Chemical context   

Pyrimidine-containing compounds exhibit various biological activities (Goldmann & Stoltefuss, 1991 ▶) and related fused heterocycles are unique classes of heterocyclic compounds that exhibit a broad spectrum of biological activities such as anti­cancer (Amin *et al.*, 2009 ▶; Pandey *et al.*, 2004 ▶), anti­viral (Ibrahim & El-Metwally, 2010 ▶), anti­bacterial (Kuyper *et al.*, 1996 ▶) and anti-oxidant (Padmaja *et al.*, 2009 ▶), anti­depressant (Kim *et al.*, 2010 ▶) and possess anti-inflammatory effects (Clark *et al.*, 2007 ▶). In addition, several piperazine derivatives have reached the stage of clinical application; among the known drugs that are used to treat anxiety is a pyrimidinylpiperazinyl compound, bu­spirone (trade name BuSpar^®^) (Tollefson *et al.*, 1991 ▶). Our research group has published a number of papers on incorporated heterocyclic ring structures, *viz.* imatinibium dipicrate (Jasinski *et al.*, 2010 ▶), 1-(2-hy­droxy­eth­yl)-4-[3-(2-tri­fluoro­methyl-9*H*-thioxanthen-9-yl­idene)prop­yl]piperazine-1,4-diium dichloride, which is the di­hydro­chloride salt of flupentixol (Siddegowda *et al.*, 2011*a* ▶) and opipramolium fumarate (Siddegowda *et al.*, 2011*b* ▶). Other related crystal structures are 4-(pyrimidin-2-yl)piperazin-1-ium (*E*)-3-carb­oxy­prop-2-enoate (Yamuna *et al.*, 2014*a* ▶), flupentixol tartarate and enrofloxacinium oxalate (Yamuna *et al.*, 2014*b* ▶,*c* ▶). As part of our ongoing studies in this area, we report herein the crystal structures of the title salts, (I) and (II).
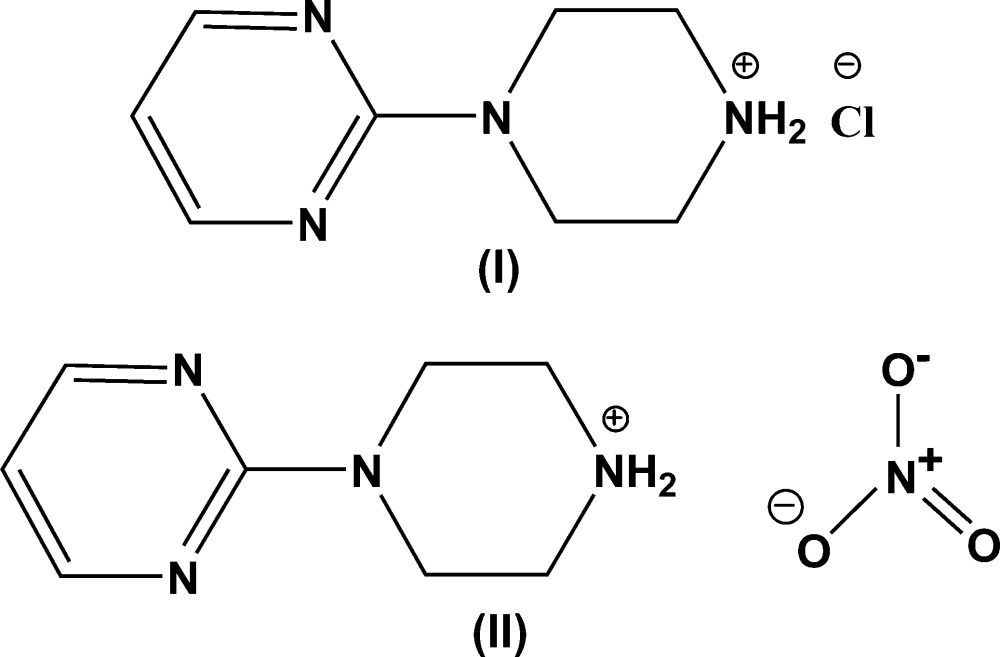


## Structural commentary   

**Figure 1:**
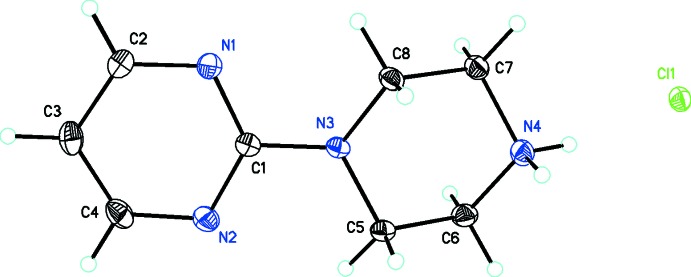
*ORTEP* drawing of C_8_H_13_N_4_^+^·Cl^−^, (I), showing 30% probability displacement ellipsoids.

**Figure 2:**
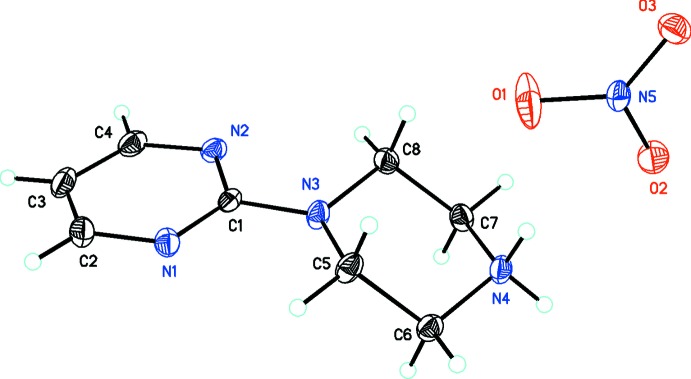
*ORTEP* drawing of C_8_H_13_N_4_^+^·NO_3_^−^, (II), showing 30% probability displacement ellipsoids.

The structure of (I) and its atom numbering are shown in Fig. 1 ▶. It consists of a pyrimidylpiperazine cation joined by the C1/N3 atoms of each unit and a chloride anion. The C1—N3 bond is 1.373 (3) Å long, which compares favorably with similar ionic structures containing this cation [1.369 (3) (Yamuna *et al.*, 2014*a* ▶), and 1.36 (6) and 1.37 (1) Å (Ding *et al.*, 2014 ▶)]. The N3/C5/C6/N4/C7/C8 piperazine unit adopts a slightly distorted chair conformation with protonation at the N4 nitro­gen atom. The structure of (II) and its atom numbering are shown in Fig. 2 ▶. Similarly, it consists of a pyrimidylpiperazine cation joined by the C1/N3 atoms of each unit and a nitrate anion. The C1—N3 bond is 1.369 (3) Å, also in the range of the related structures described above. The N3/C5/C6/N4/C7/C8 piperazine unit also adopts a slightly distorted chair conformation with protonation at the N4 atom.

## Supra­molecular features   

**Figure 3:**
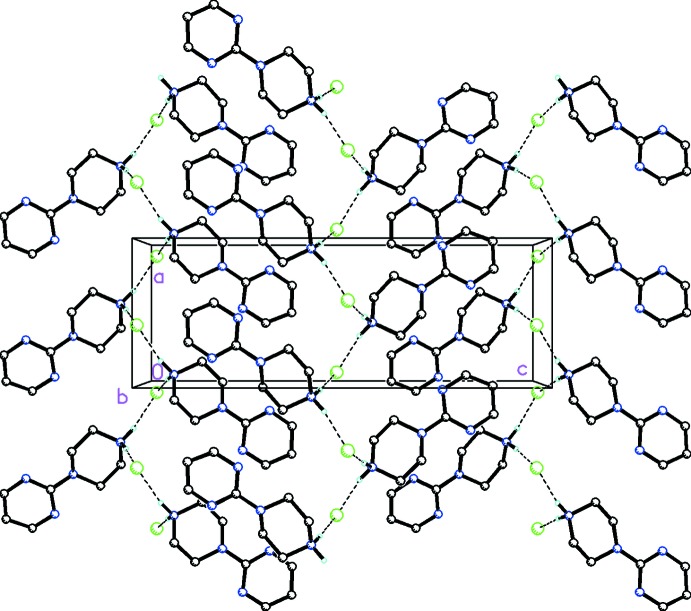
Mol­ecular packing for C_8_H_13_N_4_^+^·Cl^−^, (I), viewed along the *b* axis. Dashed lines indicate N—H⋯Cl inter­actions forming zigzag chains along the *a* axis (see Table 1 ▶ for details). H atoms not involved in hydrogen bonding have been omitted for clarity.

**Figure 4:**
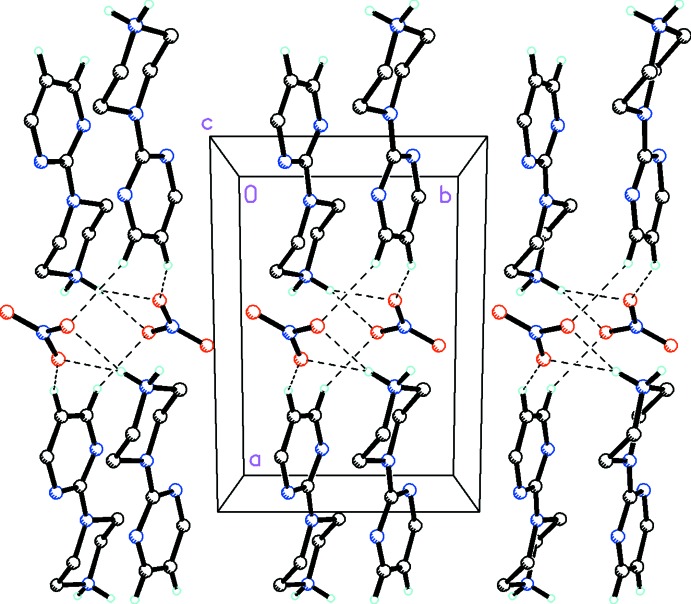
Mol­ecular packing for C_8_H_13_N_4_^+^·NO_3_^−^, (II), viewed along the *c* axis. Dashed lines indicate N—H⋯O hydrogen bonds and additional C—H⋯O inter­actions forming infinite chains along [100] (see Table 2 ▶ for details). H atoms not involved in hydrogen bonding have been omitted for clarity.

In the crystal of (I), N4—H4*A*⋯Cl1 and N4—H4*B*⋯Cl1 inter­actions are observed between pyrimidylpiperazine cations and chloride anions, forming zigzag chains along [100] (Fig. 3 ▶ and Table 1 ▶). In the crystal of (II), both of the H atoms on the N4 atom of the pyrimidylpiperazine cation are bifurcated, forming N—H⋯(O,O) hydrogen bonds (Fig. 4 ▶ and Table 2 ▶). Additional C—H⋯O inter­actions between the pyrimidyl unit and the nitrate anion are present which, in concert with the N—H⋯O hydrogen bonds between the piperazine group and nitrate anions, form infinite chains along [100].

## Database survey   

A search of the Cambridge Structural Database (Version 5.35, last update May 2014: Allen 2002 ▶) revealed only three structures containing the 4-(pyrimidin-2-yl)piperazin-1-ium cation similar to the structures reported here. These include the salts of 4-(pyrimidin-2-yl)piperazin-1-ium 3-carb­oxy­prop-2-enoate (Yamuna *et al.* 2014*a* ▶), 4-(pyrimidin-2-yl)piperazin-1-ium hydrogen d-tartrate monohydrate (Ding *et al.*, 2014 ▶) and 4-(pyrimidin-2-yl)piperazin-1-ium hydrogen l-tartrate monohydrate (Ding *et al.* 2014 ▶). The 3-carb­oxy­prop-2-enoate complex crystallizes in space group *P*2_1_/*c* while the two hydrogen (*D* and *L*)-tartrate monohydrate salts both crystallize in *P*2_1_2_1_2_1_. In comparison, title salt (I) crystallizes in *P*2_1_2_1_2_1_ while (II) crystallizes in space group *P*2_1_/*c*. In addition, as a related observation, 109 structures containing the pyrimidine–piperazine unit were also identified in this search. Some of these include, uniquely, the 4-(pyrimidin-2-yl)piperazin-1-yl unit itself. These include 1-[4-(pyrimidin-2-yl)piperazin-1-yl]ethanone, (1-methyl-1*H*-pyrrol-2-yl)[4-(pyrimidin-2-yl)piperazin-1-yl]methanone, [4-(pyrimidin-2-yl)piperazin-1-yl](2-thien­yl)methanone, (4-fluoro­phen­yl)[4-(pyrimidin-2-yl)piperazin-1-yl]methanone (Spencer *et al.*, 2011 ▶), (*E*)-1-phenyl-3-[4-(pyrimidin-2-yl)piperazin-1-yl]propan-1-one oxime (Kolasa *et al.*, 2006 ▶), *N*-(4-chloro­phen­yl)-4-(pyrimidin-2-yl)piperazine-1-carboxamide (Li, 2011 ▶) and 6-{3-[4-(pyrimidin-2-yl)piperazin-1-yl]prop­yl}-2,3-di­hydro-5*H*-[1,4]dithiino[2,3-*c*]pyrrole-5,7(6*H*)-dione (Bielenica *et al.*, 2011 ▶).

## Synthesis and crystallization   

For the preparation of title salt (I), a mixture of 1-(pyrimidin-2-yl)piperazine (0.2 g) and concentrated hydro­chloric acid (5 ml) was stirred well over a magnetic stirrer at room temperature for 10 min and then warmed at 313 K for another 10 min. A white precipitate was obtained, which was dried in the open air overnight and then dissolved in hot dimethyl sulfoxide solvent. After few days, colourless blocks were obtained on slow evaporation (m.p. above 563 K).

For the preparation of title salt (II), a mixture of 1-(pyrim­idin-2-yl)piperazine, from Sigma–Aldrich (0.2 g), and concentrated nitric acid (5 ml) was stirred well over a magnetic stirrer at room temperature for 10 min. A white precipitate was obtained immediately, which was dried in the open air overnight and then dissolved in water. After a few days, colourless blocks were obtained on slow evaporation (m.p. 463–470 K).

## Refinement   

Crystal data, data collection and structure refinement details are summarized in Table 3 ▶. In both (I) and (II), all of the H atoms were placed in their calculated positions and then refined using a riding model with C—H bond lengths of 0.93 (CH) or 0.97 Å (CH_2_) and N—H bond lengths of 0.97 Å. Isotropic displacement parameters for these atoms were set at 1.2*U*_eq_(CH,CH_2_,NH).

## Supplementary Material

Crystal structure: contains datablock(s) global, I, II. DOI: 10.1107/S1600536814020169/hb7279sup1.cif

Structure factors: contains datablock(s) I. DOI: 10.1107/S1600536814020169/hb7279Isup2.hkl

Structure factors: contains datablock(s) II. DOI: 10.1107/S1600536814020169/hb7279IIsup3.hkl

Supporting information file. DOI: 10.1107/S1600536814020169/hb7279Isup4.cml

Supporting information file. DOI: 10.1107/S1600536814020169/hb7279IIsup5.cml

CCDC references: 1023201, 1023202

Additional supporting information:  crystallographic information; 3D view; checkCIF report

## Figures and Tables

**Table 1:** Hydrogen-bond geometry (Å, °) for (I)

*D*—H⋯*A*	*D*—H	H⋯*A*	*D*⋯*A*	*D*—H⋯*A*
N4—H4*A*⋯Cl1	0.91	2.21	3.102 (2)	167
N4—H4*B*⋯Cl1^i^	0.91	2.21	3.114 (2)	175

Symmetry code: (i) 
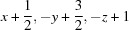
.

**Table 2:** Hydrogen-bond geometry (Å, °) for (II)

*D*—H⋯*A*	*D*—H	H⋯*A*	*D*⋯*A*	*D*—H⋯*A*
N4—H4*A*⋯O2^i^	0.91	1.92	2.829 (3)	177
N4—H4*A*⋯O3^i^	0.91	2.52	3.138 (3)	126
N4—H4*B*⋯O1	0.91	2.35	3.197 (3)	155
N4—H4*B*⋯O2	0.91	2.10	2.900 (3)	146
C3—H3⋯O1^ii^	0.95	2.46	3.240 (3)	140
C4—H4⋯O2^iii^	0.95	2.51	3.291 (3)	139

Symmetry codes: (i) 
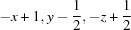
; (ii) 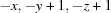; (iii) 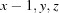.

**Table 3:** Experimental details

	(I)	(II)
Crystal data		
Chemical formula	C_8_H_13_N_4_^+^·Cl^−^	C_8_H_13_N_4_^+^·NO_3_^−^
*M*_r_	200.67	227.23
Crystal system, space group	Orthorhombic, *P*2_1_2_1_2_1_	Monoclinic, *P*2_1_/*c*
Temperature (K)	173	173
*a*, *b*, *c* (Å)	6.84764 (17), 7.27667 (18), 19.1751 (5)	10.5272 (6), 7.2230 (3), 14.1575 (7)
α, β, γ (°)	90, 90, 90	90, 107.341 (6), 90
*V* (Å^3^)	955.46 (4)	1027.58 (9)
*Z*	4	4
Radiation type	Cu *K*α	Cu *K*α
μ (mm^−1^)	3.21	0.98
Crystal size (mm)	0.26 × 0.14 × 0.08	0.22 × 0.16 × 0.06

Data collection		
Diffractometer	Agilent Agilent Eos Gemini	Agilent Agilent Eos Gemini
Absorption correction	Multi-scan (*CrysAlis RED*; Agilent, 2012 ▶)	Multi-scan (*CrysAlis RED*; Agilent, 2012 ▶)
*T*_min_, *T*_max_	0.417, 1.000	0.727, 1.000
No. of measured, independent and observed [*I* > 2σ(*I*)] reflections	5514, 1841, 1761	6218, 1960, 1752
*R*_int_	0.045	0.040
(sin θ/λ)_max_ (Å^−1^)	0.615	0.613

Refinement		
*R*[*F* ^2^ > 2σ(*F* ^2^)], *wR*(*F* ^2^), *S*	0.035, 0.091, 1.08	0.058, 0.163, 1.10
No. of reflections	1841	1960
No. of parameters	119	146
H-atom treatment	H-atom parameters constrained	H-atom parameters constrained
Δρ_max_, Δρ_min_ (e Å^−3^)	0.23, −0.20	0.42, −0.25
Absolute structure	Flack *x* determined using 687 quotients [(*I* ^+^)−(*I* ^−^)]/[(*I* ^+^)+(*I* ^−^)] (Parsons *et al.* (2013 ▶)	–
Absolute structure parameter	0.056 (15)	–

Computer programs: *CrysAlis PRO* and *CrysAlis RED* (Agilent, 2012 ▶), *SUPERFLIP* (Palatinus & Chapuis, 2007 ▶), *SHELXL2012* (Sheldrick, 2008 ▶) and *OLEX2* (Dolomanov *et al.*, 2009 ▶).

## supplementary crystallographic information

### Crystal data

**Table:**

C_8_H_13_N_4_^+^·NO_3_^−^	*F*(000) = 480
*M**_r_* = 227.23	*D*_x_ = 1.469 Mg m^−^^3^
Monoclinic, *P*2_1_/*c*	Cu *K*α radiation, λ = 1.54184 Å
*a* = 10.5272 (6) Å	Cell parameters from 2763 reflections
*b* = 7.2230 (3) Å	θ = 6.2–71.4°
*c* = 14.1575 (7) Å	µ = 0.98 mm^−^^1^
β = 107.341 (6)°	*T* = 173 K
*V* = 1027.58 (9) Å^3^	Irregular, colourless
*Z* = 4	0.22 × 0.16 × 0.06 mm

### Data collection

**Table:**

Agilent Agilent Eos Gemini diffractometer	1960 independent reflections
Radiation source: Cu Kα	1752 reflections with *I* > 2σ(*I*)
Detector resolution: 16.0416 pixels mm^-1^	*R*_int_ = 0.040
ω scans	θ_max_ = 71.0°, θ_min_ = 4.4°
Absorption correction: multi-scan (*CrysAlis RED*; Agilent, 2012)	*h* = −9→12
*T*_min_ = 0.727, *T*_max_ = 1.000	*k* = −8→8
6218 measured reflections	*l* = −17→16

### Refinement

**Table:**

Refinement on *F*^2^	Hydrogen site location: inferred from neighbouring sites
Least-squares matrix: full	H-atom parameters constrained
*R*[*F*^2^ > 2σ(*F*^2^)] = 0.058	*w* = 1/[σ^2^(*F*_o_^2^) + (0.0789*P*)^2^ + 0.9595*P*] where *P* = (*F*_o_^2^ + 2*F*_c_^2^)/3
*wR*(*F*^2^) = 0.163	(Δ/σ)_max_ < 0.001
*S* = 1.10	Δρ_max_ = 0.42 e Å^−^^3^
1960 reflections	Δρ_min_ = −0.25 e Å^−^^3^
146 parameters	Extinction correction: *SHELXL2012* (Sheldrick, 2008), Fc^*^=kFc[1+0.001xFc^2^λ^3^/sin(2θ)]^-1/4^
0 restraints	Extinction coefficient: 0.0099 (14)
Primary atom site location: structure-invariant direct methods	

### Special details

**Table:**

Geometry. All esds (except the esd in the dihedral angle between two l.s. planes) are estimated using the full covariance matrix. The cell esds are taken into account individually in the estimation of esds in distances, angles and torsion angles; correlations between esds in cell parameters are only used when they are defined by crystal symmetry. An approximate (isotropic) treatment of cell esds is used for estimating esds involving l.s. planes.

### Fractional atomic coordinates and isotropic or equivalent isotropic displacement parameters (Å^2^)

**Table:**

	*x*	*y*	*z*	*U*_iso_*/*U*_eq_	
O1	0.4119 (2)	0.6964 (4)	0.41222 (17)	0.0615 (7)	
O2	0.50951 (18)	0.6257 (2)	0.30424 (14)	0.0381 (5)	
O3	0.55020 (17)	0.8884 (2)	0.37996 (13)	0.0323 (5)	
N5	0.49103 (17)	0.7390 (3)	0.36677 (13)	0.0238 (4)	
N1	0.00592 (19)	0.2396 (3)	0.48106 (14)	0.0291 (5)	
N2	−0.11846 (18)	0.3821 (3)	0.32856 (15)	0.0273 (5)	
N3	0.10930 (18)	0.3372 (3)	0.36702 (14)	0.0268 (5)	
N4	0.33344 (18)	0.3134 (3)	0.29632 (15)	0.0278 (5)	
H4A	0.3814	0.2536	0.2617	0.033*	
H4B	0.3777	0.4191	0.3216	0.033*	
C1	−0.0049 (2)	0.3204 (3)	0.39365 (16)	0.0220 (5)	
C2	−0.1085 (3)	0.2126 (3)	0.50188 (19)	0.0346 (6)	
H2	−0.1054	0.1544	0.5627	0.042*	
C3	−0.2307 (2)	0.2647 (4)	0.4398 (2)	0.0362 (6)	
H3	−0.3111	0.2420	0.4553	0.043*	
C4	−0.2290 (2)	0.3519 (3)	0.3537 (2)	0.0329 (6)	
H4	−0.3113	0.3927	0.3097	0.039*	
C5	0.2387 (2)	0.2876 (4)	0.43489 (16)	0.0282 (5)	
H5A	0.2266	0.2035	0.4867	0.034*	
H5B	0.2848	0.4005	0.4676	0.034*	
C6	0.3222 (2)	0.1932 (3)	0.37877 (17)	0.0270 (5)	
H6A	0.4121	0.1674	0.4242	0.032*	
H6B	0.2808	0.0738	0.3519	0.032*	
C7	0.1993 (2)	0.3620 (3)	0.22801 (17)	0.0277 (5)	
H7A	0.1537	0.2483	0.1960	0.033*	
H7B	0.2095	0.4461	0.1755	0.033*	
C8	0.1166 (2)	0.4552 (3)	0.28517 (17)	0.0276 (5)	
H8A	0.1571	0.5756	0.3112	0.033*	
H8B	0.0258	0.4789	0.2407	0.033*	

### Atomic displacement parameters (Å^2^)

**Table:**

	*U*^11^	*U*^22^	*U*^33^	*U*^12^	*U*^13^	*U*^23^
O1	0.0614 (14)	0.0836 (17)	0.0562 (13)	−0.0362 (13)	0.0431 (11)	−0.0184 (12)
O2	0.0436 (10)	0.0314 (10)	0.0460 (11)	−0.0072 (8)	0.0238 (8)	−0.0153 (8)
O3	0.0379 (9)	0.0249 (9)	0.0353 (9)	−0.0045 (7)	0.0129 (7)	−0.0021 (7)
N5	0.0175 (9)	0.0301 (10)	0.0240 (9)	−0.0015 (7)	0.0064 (7)	0.0012 (8)
N1	0.0292 (10)	0.0327 (11)	0.0282 (10)	0.0020 (8)	0.0126 (8)	0.0038 (8)
N2	0.0210 (9)	0.0270 (10)	0.0331 (11)	0.0037 (7)	0.0071 (8)	0.0001 (8)
N3	0.0189 (9)	0.0380 (11)	0.0243 (9)	0.0068 (8)	0.0075 (7)	0.0087 (8)
N4	0.0231 (9)	0.0278 (10)	0.0368 (11)	−0.0042 (8)	0.0153 (8)	−0.0045 (8)
C1	0.0207 (10)	0.0220 (10)	0.0246 (11)	0.0023 (8)	0.0087 (8)	−0.0035 (8)
C2	0.0416 (14)	0.0328 (13)	0.0372 (13)	−0.0021 (11)	0.0235 (11)	−0.0014 (10)
C3	0.0300 (13)	0.0340 (13)	0.0525 (15)	−0.0049 (10)	0.0247 (11)	−0.0130 (12)
C4	0.0224 (11)	0.0304 (12)	0.0456 (15)	0.0020 (9)	0.0098 (10)	−0.0063 (11)
C5	0.0208 (11)	0.0395 (13)	0.0234 (11)	0.0087 (9)	0.0054 (9)	0.0023 (9)
C6	0.0219 (10)	0.0296 (12)	0.0293 (11)	0.0038 (9)	0.0074 (9)	0.0014 (9)
C7	0.0291 (11)	0.0305 (12)	0.0255 (11)	−0.0013 (9)	0.0111 (9)	0.0039 (9)
C8	0.0267 (11)	0.0290 (12)	0.0283 (11)	0.0033 (9)	0.0098 (9)	0.0080 (9)

### Geometric parameters (Å, º)

**Table:**

O1—N5	1.233 (3)	C2—C3	1.376 (4)
O2—N5	1.263 (2)	C3—H3	0.9500
O3—N5	1.232 (2)	C3—C4	1.377 (4)
N1—C1	1.342 (3)	C4—H4	0.9500
N1—C2	1.337 (3)	C5—H5A	0.9900
N2—C1	1.349 (3)	C5—H5B	0.9900
N2—C4	1.333 (3)	C5—C6	1.512 (3)
N3—C1	1.369 (3)	C6—H6A	0.9900
N3—C5	1.459 (3)	C6—H6B	0.9900
N3—C8	1.459 (3)	C7—H7A	0.9900
N4—H4A	0.9100	C7—H7B	0.9900
N4—H4B	0.9100	C7—C8	1.512 (3)
N4—C6	1.487 (3)	C8—H8A	0.9900
N4—C7	1.496 (3)	C8—H8B	0.9900
C2—H2	0.9500		
			
O1—N5—O2	118.2 (2)	C3—C4—H4	118.2
O3—N5—O1	121.9 (2)	N3—C5—H5A	109.7
O3—N5—O2	119.82 (18)	N3—C5—H5B	109.7
C2—N1—C1	115.6 (2)	N3—C5—C6	109.86 (18)
C4—N2—C1	115.5 (2)	H5A—C5—H5B	108.2
C1—N3—C5	121.45 (19)	C6—C5—H5A	109.7
C1—N3—C8	121.92 (18)	C6—C5—H5B	109.7
C5—N3—C8	114.01 (18)	N4—C6—C5	110.12 (18)
H4A—N4—H4B	108.0	N4—C6—H6A	109.6
C6—N4—H4A	109.4	N4—C6—H6B	109.6
C6—N4—H4B	109.4	C5—C6—H6A	109.6
C6—N4—C7	111.33 (17)	C5—C6—H6B	109.6
C7—N4—H4A	109.4	H6A—C6—H6B	108.2
C7—N4—H4B	109.4	N4—C7—H7A	109.7
N1—C1—N2	126.0 (2)	N4—C7—H7B	109.7
N1—C1—N3	116.88 (19)	N4—C7—C8	109.99 (18)
N2—C1—N3	117.06 (19)	H7A—C7—H7B	108.2
N1—C2—H2	118.3	C8—C7—H7A	109.7
N1—C2—C3	123.4 (2)	C8—C7—H7B	109.7
C3—C2—H2	118.3	N3—C8—C7	109.85 (18)
C2—C3—H3	122.1	N3—C8—H8A	109.7
C2—C3—C4	115.8 (2)	N3—C8—H8B	109.7
C4—C3—H3	122.1	C7—C8—H8A	109.7
N2—C4—C3	123.6 (2)	C7—C8—H8B	109.7
N2—C4—H4	118.2	H8A—C8—H8B	108.2
			
N1—C2—C3—C4	−1.3 (4)	C4—N2—C1—N1	−2.7 (3)
N3—C5—C6—N4	55.5 (3)	C4—N2—C1—N3	175.4 (2)
N4—C7—C8—N3	−55.3 (2)	C5—N3—C1—N1	−6.3 (3)
C1—N1—C2—C3	−0.7 (4)	C5—N3—C1—N2	175.4 (2)
C1—N2—C4—C3	0.2 (3)	C5—N3—C8—C7	56.9 (3)
C1—N3—C5—C6	141.1 (2)	C6—N4—C7—C8	56.9 (2)
C1—N3—C8—C7	−141.2 (2)	C7—N4—C6—C5	−57.0 (2)
C2—N1—C1—N2	2.9 (3)	C8—N3—C1—N1	−166.9 (2)
C2—N1—C1—N3	−175.2 (2)	C8—N3—C1—N2	14.8 (3)
C2—C3—C4—N2	1.6 (4)	C8—N3—C5—C6	−57.0 (3)

### Hydrogen-bond geometry (Å, º)

**Table:**

*D*—H···*A*	*D*—H	H···*A*	*D*···*A*	*D*—H···*A*
N4—H4*A*···O2^i^	0.91	1.92	2.829 (3)	177
N4—H4*A*···O3^i^	0.91	2.52	3.138 (3)	126
N4—H4*B*···O1	0.91	2.35	3.197 (3)	155
N4—H4*B*···O2	0.91	2.10	2.900 (3)	146
C3—H3···O1^ii^	0.95	2.46	3.240 (3)	140
C4—H4···O2^iii^	0.95	2.51	3.291 (3)	139

Symmetry codes: (i) −*x*+1, *y*−1/2, −*z*+1/2; (ii) −*x*, −*y*+1, −*z*+1; (iii) *x*−1, *y*, *z*.
